# Modulation of the Antioxidant System of Caco-2 Cells in the Presence of Aflatoxin B1, Ochratoxin A, and Ferulic Acid

**DOI:** 10.3390/toxins17060274

**Published:** 2025-05-30

**Authors:** Andreea-Luminița Rădulescu, Roua Gabriela Popescu, Mihaela Balas, George Cătălin Marinescu, Anca Dinischiotu

**Affiliations:** 1 Department of Biochemistry and Molecular Biology, Faculty of Biology, University of Bucharest, 050095 Bucharest, Romania; a.radulescu20@s.bio.unibuc.ro (A.-L.R.); mihaela.balas@bio.unibuc.ro (M.B.); 2 Independent Research Association, 012416 Bucharest, Romania; roua@independent-research.ro (R.G.P.); catalin.marinescu@independent-research.ro (G.C.M.); 3 Blue Screen SRL, 012416 Bucharest, Romania

**Keywords:** ochratoxin A, aflatoxin B1, ferulic acid, oxidative stress, Nrf2

## Abstract

Food security and food safety are major aspects for human and animal health, yet mycotoxins contaminate 60–80% of food crops before and after harvest, elevating the risk of chronic toxicity and cancer development. This study investigates the potential of ferulic acid (FA) as an antioxidant against mycotoxin-induced oxidative stress in Caco-2 cells exposed to aflatoxin B1 (AFB1) and ochratoxin A (OTA) for 24 and 48 h. The effects on the degree of lipid peroxidation and non-enzymatic and enzymatic mechanisms against oxidative stress were evaluated. FA appears to mitigate oxidative stress by modulating lipid and protein oxidation, decreasing the level of 4-hydroxy-2-nonenal (4-HNE), increasing superoxide dismutase (SOD) activity, and preserving thiol groups by scavenging reactive oxygen species (ROS). Additionally, the reduction in polyubiquitinated Nrf2 level, and higher SOD activity, suggest that FA stabilizes Nrf2, delaying its degradation and reinforcing its antioxidant role. These findings indicate that FA partially counteracts mycotoxin-induced oxidative damage, highlighting the need for further investigation into its long-term effects.

## 1. Introduction

Food security and food safety are aspects of great importance for food systems with important implications for human and animal health [1,2]. Food safety requires strict regulations regarding the acceptable limits of different toxins and toxicants in food and feed [3], both natural and synthetic. Natural toxins are produced by living organisms and are toxic to humans and animals. The most frequent natural toxins found in food are mycotoxins, aquatic biotoxins, cyanogenic glycosides, furocoumarins, lectins, solanines, chaconine, etc. [4].

Mycotoxins are secondary metabolites of filamentous fungi, produced before and after harvest as well as during the manipulation and storage of cereals, oilseeds, dried fruits, coffee, medicinal plants, etc. [5]. The most important fungal genera of these are *Aspergillus*, *Penicillium*, *Alternaria*, *Fusarium*, and *Claviceps* [6]. Each fungal species may produce several types of mycotoxins, as well as different species to generate them is very probable. It was estimated that all over the world, about 60–80% of food crops are contaminated with these [2]. From a chemical point of view, mycotoxins can be classified into major groups such as trichothecenes, zearalenones, ochratoxins, aflatoxins, and fumonisins, as well as emerging or less frequently reported toxins including enniatins, beauvericin, patulin, moniliformin, and fusaric acid [7].

In the conditions of climate change, rising temperature, water scarcity, severe droughts, forest fires, and heavy rains, the alteration of the geographical distribution of foodborne pathogens occurs. Mycotoxins’ biosynthesis depends on temperature and humidity. Different fungi have variable development conditions regarding temperature and relative humidity, and consequently, climate change impacts them in a variable way [8]. Also, climate change influences the geographical distribution of mycotoxigenic fungal species. This generates competition between species of the same genus or different ones, resulting in changes in the main contaminant mycotoxins of the crops [9,10].

Mycotoxins’ chronic exposure in animals and humans determines genotoxicity, teratogenicity, neurotoxicity, hepatotoxicity, immunotoxicity, gastrointestinal toxicity, nephrotoxicity, and sometimes, cancer development [11].

Aflatoxins (AFs) are especially present in maize and cause hepatitis and liver cancer [12] and severely affect growth and development [13]. They are produced by several species of *Aspergillus*. The best-known AFs are B1, B2, G1, G2, M1, and M2; AFB1 is the most toxic carcinogen.

Ochratoxins are produced by the *Aspergillus* and *Penicillium* genera and are represented by more than 20 subtypes, with ochratoxin A (OTA) being the most toxic [14].

Human exposure to OTA occurs principally in Europe and Canada, where barley and wheat are used extensively in bakery products. The kidney is the major target organ for this mycotoxin [15]; this is also associated with chronic tubule-interstitial kidney disease, i.e., Balkan endemic nephropathy [16].

Due to their chemical structures, mycotoxins are resistant to high temperatures, and generally, they cannot be inactivated during food preparation. Consequently, several mitigation strategies have been developed to diminish their toxic effects in humans and animals. These are chemical (addition of acid, alkaline, and oxidant reagents), physical (UV treatment, electromagnetic field, cold plasma treatment, etc.), and biological (enzymes or microorganisms) [7,17]. All these approaches can be effective, but generate concerns regarding the safety of reaction end products and maintenance of the nutritional and organoleptic qualities. Consequently, nutritional approaches, such as the constant intake of antioxidants, became an important option for human and animal nutrition, considering that exposure to mycotoxins is unavoidable [18].

Previously, it was shown that the toxic effect of major mycotoxins is mediated by oxidative stress, which occurs when an imbalance between the reactive oxygen species (ROS) level and the antioxidant defense system capacity exists. Consequently, the peroxidation of polyunsaturated fatty acids, as well as oxidative damage to DNA and proteins, are observed upon human and animal exposure to mycotoxins. In this context, the intake of natural antioxidants is useful to diminish these toxic effects [19].

Polyphenols are organic compounds synthesized by the secondary metabolism of plants, with antioxidant and anti-inflammatory properties due to their ability to inhibit reactive oxygen and nitrogen species, by direct interaction and/or activation of antioxidant enzymes [20]. They can be classified as flavonoids (flavones, flavanols, isoflavones, anthocyanidins, and anthocyanins) and non-flavonoids. All flavonoids present a diphenyl propane structure in which phenolic rings are linked by a heterocyclic ring, usually pyran. Non-flavonoids are represented by phenolic acids, stilbenes, and lignans and contain a single aromatic ring with different numbers and locations of hydroxyl groups. Phenolic acids can be divided depending on the parent skeleton into hydroxycinnamic and hydroxybenzoic ones. In vegetables and fruits, ferulic, caffeic, p-coumaric, and sinapic acids are the most common hydroxy cinnamic acids [21].

Ferulic acid (FA) (4-hydroxy-3-methoxy cinnamic acid) has scavenger properties due to the electron donating groups on the aromatic ring, the presence of an unsaturated C-C double bond adjacent to the carboxyl group that could attack sites of free radicals, and the potential capacity of carboxyl group to interact electrostatically with phosphatidyl choline and phosphatidyl ethanolamine residues of the membranes, preventing lipid peroxidation [22].

FA capacity to mitigate oxidative stress was demonstrated in several studies using cellular models like hepatocytes [23,24], β-cells in the pancreas [25,26,27,28], cardiac cells [23], muscle fibers [29,30], neurons [31], lymphocytes [32], erythrocytes [33], Leydig cells [34], gonads [35], and embryonic cells [30,36]. Also, a study on rat cells revealed the efficiency of FA in the protection of the duodenal barrier against damage to the duodenal villi, rupture of the tight junctions, and fracture of microvilli induced by AFB1 exposure [37].

As far as we know, FA was used to counteract only the toxic effects of trichothecenes on animal cells [38]. Considering this, the aim of our paper was to study the toxic effects induced by the combined exposure to AFB1 and OTA in Caco-2 cells clone C2BBe1 and the capacity of FA to counteract these.

## 2. Results

**Figure A1:**
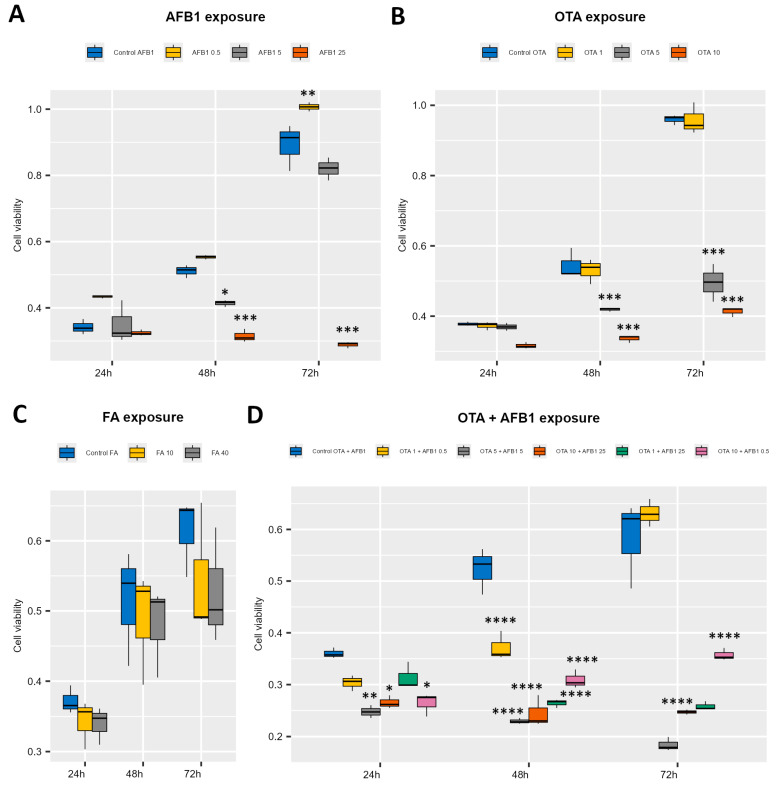
Evaluation of cell viability by MTT assay following single exposure of C2BBe1 cells to different concentrations of aflatoxin B1 (AFB1) (**A**), ochratoxin A (OTA) (**B**), and ferulic acid (FA) (**C**), and in combined exposure of OTA + AFB1 (**D**) for 24, 48, and 72 h. In legend, all compound addition numbers represent the concentration as μM. The statistical significance highlighted is against the Control group at the corresponding time, where * *p* < 0.05; ** *p* < 0.01; *** *p* < 0.001; **** *p* < 0.0001.

Firstly, cell viability was assessed for OTA, AFB1, and FA as single compound exposure, at 3 time intervals, by MTT assay. Therefore, the following concentrations were used for AFB1 [0.1, 0.5, 1, 5, 7.5, 10, 25, 50, 75, 100, and 150 μM], OTA [0.05, 0.1, 0.5, 1, 5, 10, 20, 30, 40, 50, and 60 μM], and FA [0.5, 5, 10, 20, 40, 50, 100, 200, 300, 400, and 500 μM]. Following the evaluation of cell viability at the three exposure time intervals (24, 48, and 72 h), 3 concentrations for each compound were chosen from the initial 11 concentrations: AFB1 [0.5, 5, and 25 μM] (Figure A1A), OTA [1, 5, and 10 μM] (Figure A1B), and FA [10 and 40 μM] (Figure A1C).

**Figure A2:**
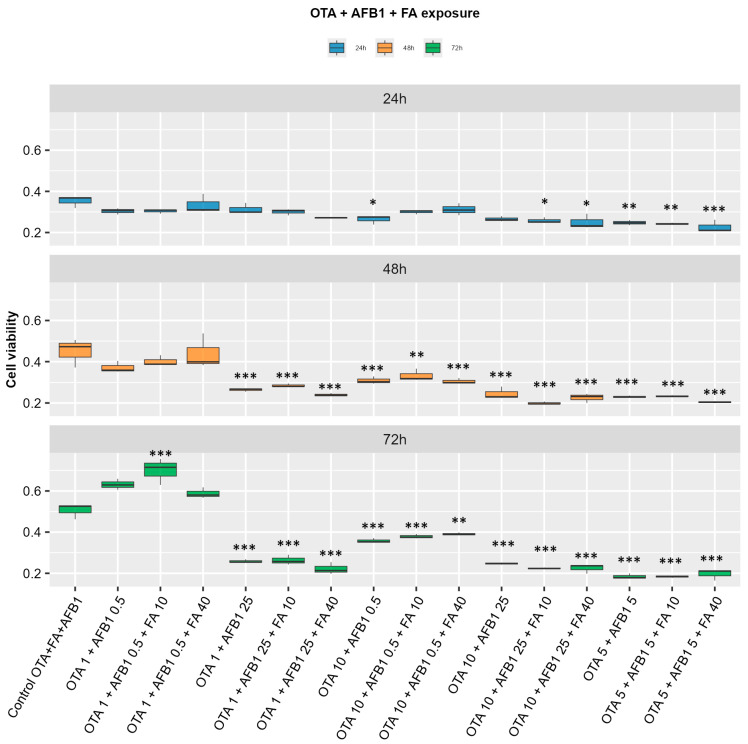
Evaluation of cell viability by MTT assay following exposure of C2BBe1 cells to the combination of AFB1, OTA, and FA at different concentrations for 24, 48, and 72 h. On the OX axis, the concentration combinations are highlighted, where the additional number of each compound represents the concentration in μM. The statistical significance highlighted is against the Control group at the corresponding time, where * *p* < 0.05; ** *p* < 0.01; *** *p* < 0.001.

After that, combinations of mycotoxin concentrations (OTA and AFB1) of the previously chosen ones were realized; thus, among all possible concentrations, only 5 combinations were tested (Figure A1D). In addition, the 5 combinations of AFB1 + OTA were also tested in the presence of FA in concentrations of 10 and 40 μM (Figure A2). All concentrations used in this study are listed in Table A1. Following the statistical analysis with a two-way ANOVA test and comparative analysis against the control group (Tukey) corresponding to the time point of exposure, the observed changes in cell viability are significant, with a power > 0.89 at the 0.05 significance level. Thus, following evaluation of the cell viability of the combinations with all three compounds, the combination of 25 µM AFB1 + 10 µM OTA + 10 µM FA was chosen for the following tests.

In terms of exposure intervals, it was observed that at 72 h of exposure with one, two, or a combination of three compounds, cell viability decreased by more than 50%. Thus, for evaluating the degree of lipid peroxidation and non-enzymatic and enzymatic mechanisms involved in combating oxidative stress, only two exposure time intervals were maintained, 24 and 48 h, respectively.

### 2.1. Indicators of Oxidative Stress and Lipid Peroxidation

**Figure 1:**
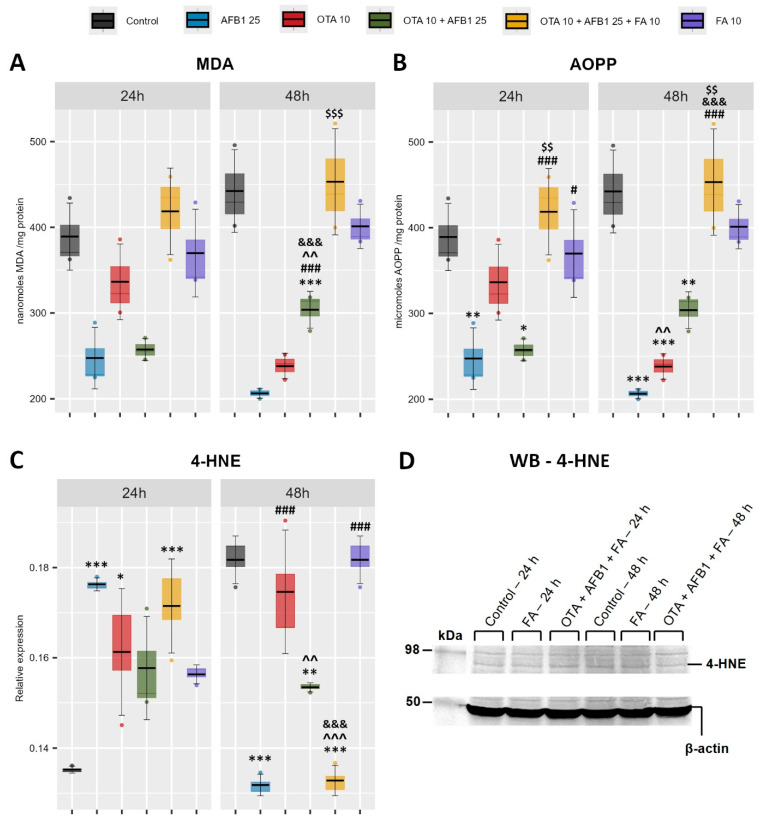
Evaluation of oxidative stress and lipid peroxidation by analysis of malondialdehyde (MDA) (**A**), advanced oxidation protein products (AOPP) (**B**), 4-hydroxynonenal (4-HNE) (**C**) levels, and (**D**) Western blot for 4-HNE, following exposure of C2BBe1 cells to ochratoxin A (OTA), aflatoxin B1 (AFB1), and ferulic acid (FA). Results are presented as means ± standard deviation (SD) in six experimental conditions (Control; AFB1 25 μM; OTA 10 μM; OTA 10 μM + AFB1 25 μM; OTA 10 μM + AFB1 25 μM FA 10 μM) at 24 h and 48 h. The statistical significance is shown against the Control group—*, against the AFB1 25 μM group—#, against the OTA 10 μM group—&, against the FA 10 μM group—^, against the OTA 10 μM + AFB1 25 μM group—$, at the corresponding exposure time, where */# *p* < 0.05; **/^^/$$ *p* < 0.01; ***/###/^^^/&&&/$$$ *p* < 0.001.

As oxidative stress following mycotoxin exposure can lead to lipid peroxidation, which damages cell membranes and disrupts cellular functions, the following was assessed: malondialdehyde (MDA) and 4-hydroxynonenal (4-HNE) levels, as markers of lipid peroxidation and advanced oxidation protein products (AOPP), as an indicator of protein oxidation their levels being associated with oxidative stress and cell damage (Figure 1).

The MDA level (Figure 1A) is significantly decreased only at 48 h in AFB1 + OTA co-exposure compared to the Control but also significantly increased in the condition where ferulic acid is added together with mycotoxins (AFB1 + OTA + FA) compared to that without ferulic acid (AFB1 + OTA). Also, by evaluating AOPP (Figure 1B), a similar trend as in MDA was observed, in which the level of AOPP is significantly decreased in the AFB1 + OTA group compared to the Control group, and a significant increase in the AFB1 + OTA + FA group compared to the group without FA (AFB1 + OTA), but not only at 48 h but also at 24 h. In addition, a significant decrease in AOPP was observed in the AFB1 group compared to the Control group at both exposure times, but more pronounced at 48 h.

Very interestingly observed is the level of 4-HNE (Figure 1C), significantly different at 24 h compared to 48 h. Thus, if at 24 h, the level of 4-HNE is significantly increased in the AFB1, OTA, and AFB1 + OTA + FA groups; at 48 h, the level of 4-HNE in the AFB1, OTA, and AFB1 + OTA + FA groups is significantly decreased compared to the Control group. Moreover, at 48 h, the level decreases significantly in the triple combination group (AFB1 + OTA + FA) compared to the group without ferulic acid (AFB1 + OTA) but also compared to the group exposed to ferulic acid only (FA).

### 2.2. Nrf2 Activation as Response to Oxidative Stress

**Figure 2:**
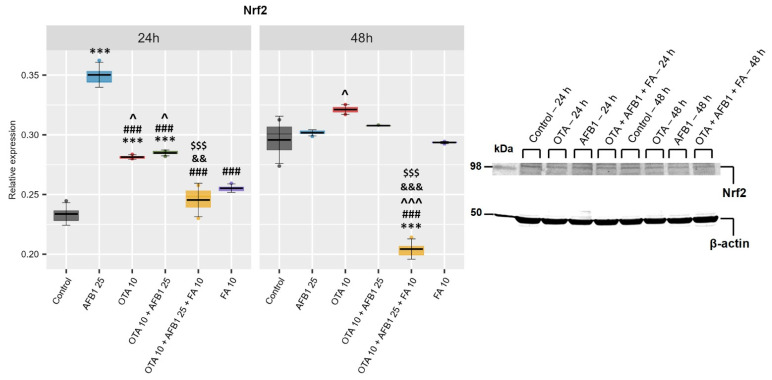
Evaluation of nuclear factor erythroid 2-related factor 2 (Nrf2) relative protein expression as a response to oxidative stress, following exposure of C2BBe1 cells to ochratoxin A (OTA), aflatoxin B1 (AFB1), and ferulic acid (FA). Results are presented as means ± standard deviation (SD) in six experimental conditions (Control; AFB1 25 μM; OTA 10 μM; OTA 10 μM + AFB1 25 μM; OTA 10 μM + AFB1 25 μM FA 10 μM) at 24 h and 48 h. The statistical significance is shown against the Control group—*, against the AFB1 25 μM group—#, against the OTA 10 μM group—&, against the FA 10 μM group—^, against the OTA 10 μM + AFB1 25 μM group—$, at the corresponding exposure time, where ^ *p* < 0.05; && *p* < 0.01; ***/###/^^^/&&&/$$$ *p* < 0.001.

Nuclear factor erythroid 2-related factor 2 (Nrf2) is a key transcriptional factor that regulates cellular antioxidant defense mechanisms; therefore, the presence of mycotoxins could cause Nrf2 activation. By evaluation of relative protein expression by Western Blot (Figure 2), we observed a significant increase in Nrf2 levels in the mycotoxin groups (AFB1, OTA, AFB1 + OTA) compared to the Control group. In the group in which ferulic acid is added together with mycotoxins (AFB1 + OTA + FA), a significant decrease was observed compared to the Control group at 48 h. In addition, a significant decrease was observed in the triple combination group (AFB1 + OTA + FA) compared to the AFB1, OTA, and combined (AFB1 + OTA) groups at both exposure times.

### 2.3. Non-Enzymatic Antioxidant Defense

**Figure 3:**
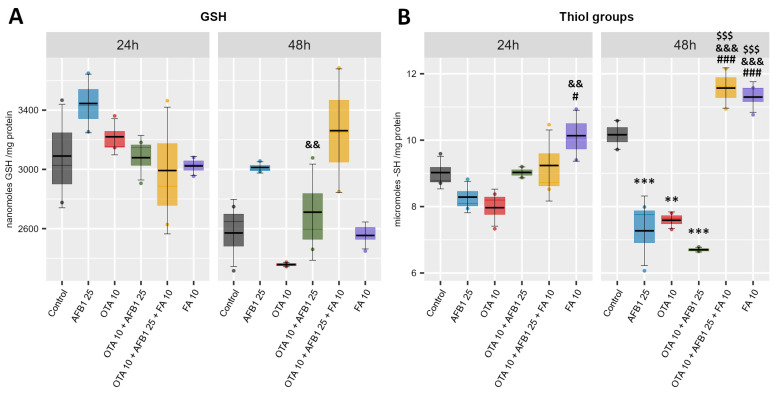
Evaluation of non-enzymatic antioxidant defense by analysis of glutathione (GSH) (**A**) and thiol groups (**B**) levels, following exposure of C2BBe1 cells to ochratoxin A (OTA), aflatoxin B1 (AFB1), and ferulic acid (FA). Results are presented as means ± standard deviation (SD) in six experimental conditions (Control; AFB1 25 μM; OTA 10 μM; OTA 10 μM + AFB1 25 μM; OTA 10 μM + AFB1 25 μM FA 10 μM) at 24 h and 48 h. The statistical significance is shown against the Control group—*, against the AFB1 25 μM group—#, against the OTA 10 μM group—&, against the OTA 10 μM + AFB1 25 μM group—$, at the corresponding exposure time, where # *p* < 0.05; **/&& *p* < 0.01; ***/###/&&&/$$$ *p* < 0.001.

Glutathione (GSH) is the most abundant intracellular non-enzymatic antioxidant, its concentration being critical in detoxification processes, response, or resistance to oxidative stress as it acts as a redox buffer by donating electrons to neutralize reactive oxygen species (ROS). Also, the content of thiol (-SH) groups in proteins and molecules play a crucial role in intracellular redox balance. Thus, the level of GSH and the content of thiol groups in the five experimental groups (AFB1 25 μM; OTA 10 μM; OTA 10 μM + AFB1 25 μM; OTA 10 μM + AFB1 25 μM FA 10 μM) were evaluated in comparison with the Control group (Figure 3).

Regarding the GSH level (Figure 3A), a significant increase was observed only in the mycotoxin combination group (AFB1 + OTA) compared to the OTA group at 48 h. Also, the thiol groups content (Figure 3B) is significantly decreased in the mycotoxin groups (AFB1, OTA, AFB1 + OTA) compared to the Control group at 48 h, the decrease being more pronounced in the combined group (AFB1 + OTA) compared to the single exposure groups (AFB1 and OTA). However, a significant increase was observed in the mycotoxin group supplemented with ferulic acid (AFB1 + OTA + FA) compared to the mycotoxin groups (AFB1, OTA, AFB1 + OTA) at 48 h. In addition, the level of thiol groups is significantly increased in the ferulic acid (FA) group compared to the mycotoxin groups at both exposure times.

### 2.4. Enzymatic Antioxidant Defense

**Figure 4:**
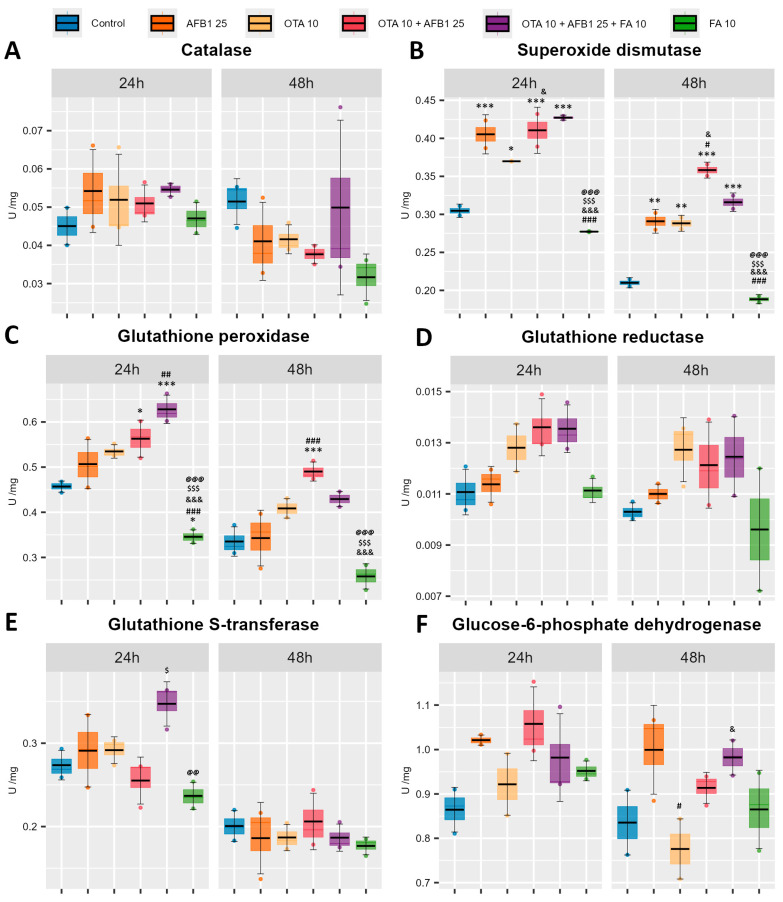
Evaluation of the enzymatic antioxidant defense by analyzing the specific activity (U/mg) of catalase (**A**), superoxide dismutase (**B**), glutathione peroxidase (**C**), glutathione reductase (**D**), glutathione S-transferase (**E**), and glucose-6-phosphate dehydrogenase (**F**) in C2BBe1 cells following exposure to ochratoxin A (OTA), aflatoxin B1 (AFB1), and ferulic acid (FA). Results are presented as means ± standard deviation (SD) in six experimental conditions (Control; AFB1 25 μM; OTA 10 μM; OTA 10 μM + AFB1 25 μM; OTA 10 μM + AFB1 25 μM FA 10 μM) at 24 h and 48 h. The statistical significance is shown against the Control group—*, against the AFB1 25 μM group—#, against the OTA 10 μM group—&, against the OTA 10 μM + AFB1 25 μM group—$, against the OTA 10 μM + AFB1 25 μM + FA 10 μM group—@, at the corresponding exposure time, where */#/&/$ *p* < 0.05; **/##/@@ *p* < 0.01; ***/###/&&&/$$$/@@@ *p* < 0.001.

Antioxidant defense mechanisms play a crucial role in maintaining cellular redox balance by neutralizing ROS and preventing oxidative damage. Therefore, to visualize from a different point of view the impact of AFB1, OTA and FA on the antioxidant capacity of C2BBe1 cells, the specific activities for catalase (CAT), superoxide dismutase (SOD), glutathione peroxidase (GPX), glutathione reductase (GR), glutathione S-transferase (GST), and glucose-6-phosphate dehydrogenase (G6PDH) were evaluated (Figure 4).

No significant changes were observed for CAT (Figure 4A) and GR (Figure 4D) in none of the comparisons at the corresponding exposure time.

The specific activity of superoxide dismutase (SOD) (Figure 4B) is significantly increased in the mycotoxin groups (AFB1, OTA, AFB1 + OTA) and the ferulic acid-supplemented group (AFB1 + OTA + FA) compared to the Control group at both exposure times. Also, the increase in specific activity in the combined mycotoxin group (AFB1 + OTA) compared to the mycotoxin alone groups (AFB1, OTA) is significantly increased. Furthermore, in the ferulic acid (FA) group, a significant decrease in SOD specific activity is observed compared to the mycotoxin groups (AFB1, OTA, AFB1 + OTA), but also in the mycotoxin and ferulic acid (AFB1 + OTA + FA) group, at both exposure times. This suggests an increased enzymatic substrate level, i.e., superoxide anion.

In a similar manner to SOD specific activity, glutathione peroxidase specific activity (Figure 4C) in the ferulic acid (FA) group decreases significantly at 24 h and 48 h compared to the OTA, AFB1 + OTA, and AFB1 + OTA + FA groups, but in addition at 24 h, specific activity also decreases compared to the Control and AFB1 groups. Moreover, in the AFB1 + OTA group, a significant increase is observed at both exposure times, compared to the Control group, and at 48 h, it is also significant compared to the AFB1 group. Also, at 24 h, the AFB1 + OTA + FA group shows a significantly increased specific glutathione peroxidase activity compared to the Control and AFB1 groups.

Glutathione S-transferase (GST) specific activity (Figure 4E) is significantly changed only at 24 h. It significantly decreases in the group with ferulic acid (FA) compared to the group with ferulic acid and the two mycotoxins (AFB1 + OTA + FA) and significantly increases in the AFB1 + OTA + FA group compared to the group without ferulic acid (AFB1 + OTA), but only with the two mycotoxins in combination.

Glucose-6-phosphate dehydrogenase (G6PDH) specific activity (Figure 4F) is significantly modified only at 48 h, where a significant decrease is observed in the OTA group compared to the AFB1 group and a significant increase in the AFB1 + OTA + FA group compared to the OTA group.

## 3. Discussion

Previously, concomitant exposure to OTA and AFB1 has been studied on human liver, immune, kidney, neuronal, testicular, and intestinal cells [39]. This study is the first to address the effect of pure FA for modulating oxidative stress in Caco-2 cells induced by simultaneous exposure to AFB1 and OTA.

The gastrointestinal tract represents the first physical and chemical barrier to exogenous compounds introduced by ingestion. The absorption, mainly occurring at the intestinal level, is the step that establishes the xenobiotic fraction that ends up in the blood. The intestinal epithelium is represented by a single layer comprising enterocytes that are predominant, Goblet cells, and Paneth cells. The enterocytes are held together by tight junctions and present thousands of microvilli over apical membranes, known as the brush border, implicated in absorption. These cells contain xenobiotic- and food-metabolizing enzymes that decrease the oral bioavailability of xenobiotics, i.e., the dose of intact compound that reaches the systemic circulation [40]. In fact, gut epithelial cells represent interfaces between the host and environment [41].

In this study, we used the Caco-2-BBE1 clone cells because they form a polarized monolayer with an apical brush border like human colon cells [42] and apical junctions, establishing the paracellular barrier.

In the intestine, more than 80% of AFB1 is absorbed after ingestion [43]. Also, the oral bioavailability of OTA is about 93% in humans, 60% in pigs, and lower in rodents [44], being absorbed from the stomach and jejunum [45].

Previously, it was proved that AFB1 exposure induced an increased ROS level [46], just like OTA [47] in porcine intestinal epithelial cells. A long time ago, it was proved that AFB1 exposure decreased the activity of NADH-cytochrome C reductase complex in hepatocytes [48], and more recently, it was demonstrated that this disrupted the normal mitochondrial lipid metabolism via COX-2 and mitophagy pathway [49]. On the other hand, OTA treatment induced loss of mitochondrial potential and decreased the cellular ATP concentration [50] and generated ROS in hepatocytes [51].

The phospholipids from the mitochondrial inner membrane interact directly with protein complexes I-IV, modulating their function. Having a hydrophobic structure, both AFB1 and OTA could interact with these phospholipids, and as a result, the tridimensional structure of the proteins of these complexes could be affected. Consequently, the leak of electrons on their way to molecular oxygen along the mitochondrial electron transport chain, at the level of complexes I and III, could increase, generating superoxide anion.

In our experiments, probably even though enterocytes have fewer mitochondria compared to hepatocytes [52], being specialized in absorption, the level of superoxide increased significantly after 24 h and 48 h, in the samples treated with OTA, AFB1, and OTA + AFB1. Consequently, total SOD activity also rose significantly due to the substrate excess. As can be seen in Figure 4B, an action synergy between the two mycotoxins occurred and total SOD activity was higher, especially after 48 h in the samples treated concomitantly with both mycotoxins.

FA, being a polyphenolic compound, could undergo repeated oxidation and reduction reactions in the presence of AFB1 and OTA and participate in Michael addition reactions with cysteine residues on Kelch-like ECH-associated protein 1 (Keap1) [53], which determines the activation of Nrf2 transcription factor, which is involved in the cellular xenobiotic and oxidative stress response. It is highly regulated by ubiquitylation and proteasomal degradation [54]. Considering that we used an antibody against polyubiquitylated Nrf2, the decrease in its expression in the sample exposed to AFB1 + OTA + FA compared to AFB1 + OTA one could suggest that a higher quantity of transcription factor migrated in the nucleus and up-regulated the expression of genes that codify for antioxidant enzymes and detoxification proteins [55], especially SOD and GPX isoenzymes. On the other hand, FA presents a weaker superoxide anion scavenging activity compared with other non-enzymatic antioxidants such as epigallocatechin gallate and ascorbic acid [56].

Possibly, after 24 h exposure, the weak increase in total SOD activity in AFB1 + OTA + FA condition compared to AFB1 + OTA one could be due to the increase in SOD isoenzymes expression. Also, the decrease of this after 48 h could occur because in a longer time, FA interacted with superoxide, diminishing the level of substrate for the SOD isoenzymes. Both Cu, Zn-SOD and Mn-SOD catalyze the dismutation reaction of superoxide and protons, generating hydrogen peroxide and molecular oxygen.

The hydrogen peroxide is decomposed in water and molecular oxygen in the reaction catalyzed by two antioxidant enzymes: CAT and GPX. These enzymes have different Michaelis constant (K_M_) values for hydrogen peroxide. CAT has a higher K_M_, in different systems ranging from high μM to low mM [57], and a low affinity for substrate, and consequently, it is active when high quantities of hydrogen peroxide are present in the cells. GPX catalyzes the transformation of hydrogen peroxide or lipid peroxides in the presence of GSH in water, respectively lipid hydroxyls, and has a lower K_M_ for hydrogen peroxide_,_ being active when lower quantities of substrate exist. Also, GPX affinity for hydrogen peroxide is greater than for lipid peroxides [58]. Analyzing Figure 3A and Figure 4C, we could conclude that the decrease in hydrogen peroxide level took place in the reaction catalyzed by GPX.

On the other hand, superoxide reacts with hydrogen peroxide according to the Haber Weiss reaction, generating hydroxyl radical, which is a short-lived molecule, that attacks the biomolecules situated at a few nanometers distance, in a non-specific way. The most sensitive to this attack are polyunsaturated fatty acids (PUFAs). Hydroxyl radical extracts a hydrogen atom from a carbon–carbon double bond, and then an oxygen insertion occurs, resulting in lipid peroxyl radicals and hydroperoxides. The end-products of peroxidation of arachidonic acid and other long-chain PUFAs, such as linoleic and γ-linolenic acids, include MDA [59] and 4-HNE [60]. Up to 24 h of exposure, glutathione peroxidase activity succeeded in removing MDA in all experimental conditions. Later, the concomitant exposure to AFB1 and OTA generated such an important quantity of MDA that glutathione peroxidase could not diminish this compound enough.

Other enzymes involved in the detoxification of lipid peroxides are GSTs, which catalyze the conjugation of activated xenobiotics with GSH, resulting in the formation of oxidized glutathione [61]. As shown in Figure 4E, total GST activity did not vary after 24 h and 48 h in all conditions except the sample treated with AFB1 + OTA + FA for 24 h. This suggests that GSTs were not involved in the conjugation of MDA, 4-HNE, and/or AFB1 and OTA, but possibly after 24 h of treatment, FA stimulated the biosynthesis of some isoforms of GSTs. This could be correlated with the slight decrease in MDA level after 24 h in this sample compared to that of AFB1 + OTA. After 48 h, the significant increase in the sample exposed to AFB1 + OTA could be correlated with the increase in glutathione peroxidase activity, suggesting that the Caco-2-BBe1 clone cells tried to counteract the generation of MDA.

Moreover, 4-HNE is an electrophilic compound that is highly reactive toward nucleophilic thiol and amino groups. It can react with histidine, cysteine, and lysine residues of proteins, contributing to protein cross-linking [62]. The addition of FA in the AFB1 + OTA + FA group appears to prevent excessive lipid peroxidation by reducing the level of 4-HNE already formed (Figure 1C). Interestingly, while MDA continued to accumulate, 4-HNE levels showed a biphasic response. In the early phase of exposure, i.e., at 24 h, FA acts as a pro-oxidant [63], accelerating lipid peroxidation processes, leading to an increase in 4-HNE levels at 24 h, and significantly reduced in the late phase, at 48 h, where FA leads to activation of the antioxidant defense system, including increased SOD activity and activation of downstream antioxidant systems (GPX and GSH) (Figure 4), which increases the rate of scavenging and neutralization of the initially formed 4-HNE, explaining the biphasic response, with increased 4-HNE in the early phase and decreased 4-HNE in the late phase. More specifically, in this late phase, a significant rise of SOD activity is observed in the AFB1 + OTA + FA group, which probably leads to a reduction in the level of superoxide anion (O_2_^−^) formed, including the generation of secondary radicals, such as 4-HNE [64]. This adaptation could explain the observed 4-HNE drop.

Although FA leads to reduced lipid peroxidation by lowering 4-HNE levels, protein oxidation is upregulated by rising AOPP levels, as shown in Figure 1B. AOPPs are formed when proteins undergo oxidative modifications, mainly due to ROS and chlorinated oxidants, such as chloramines and hypochlorous acid (HOCl) [65]. The significant increase in AOPP levels in the AFB1 + OTA + FA group at both 24 h and 48 h may suggest that OTA and AFB1 may induce stress in different cellular compartments, while FA can stimulate the biosynthesis of ROS scavenging enzymes through interactions with Nrf2. This results in a redistribution of oxidative damage, shifting stress from lipid to protein, altering the redox balance or proteasomal degradation pathways in a way that protects cell membranes but leaves proteins more vulnerable to oxidation, as protein repair systems are slower [66,67]. As previously observed [63], while antioxidant enzyme activities (such as SOD and G6PDH) are raised in response to oxidative stress, this increase may be not sufficient to immediately neutralize all ROS, particularly in the early phases when ROS levels exceed detoxification capacity.

GSH is a common substrate for GPX and GST. It is a tripeptide, γ-glutamyl-cysteinyl--glycine, which is the most important non-enzymatic antioxidant in mammalian cells. The sulfhydryl (-SH) group of cysteine is implicated in the reduction and conjugation reactions. So, GSH can form adducts with xenobiotics in a non-enzymatic way or in a reaction catalyzed by GST. Moreover, it is used in the elimination of electrophiles like MDA. Still, most of the GSH is utilized by GPX isoforms, which reduce peroxides and maintain redox homeostasis [68].

Our data revealed a rise in GSH level in OTA-exposed cells compared to the control, which is in contrast with that of Schaaf et al. [69] in proximal tubular cells. Also, exposure of HepG2 cells to OTA 10 μM did not affect the GSH level [70]. The significant increase in GSH level after 48 h of treatment with AFB1 + OTA + FA could be due to the up-regulation of γ-glutamyl-cysteine ligase and glutathione synthetase, enzymes implicated in GSH biosynthesis as well as in that of glutathione reductase, even though this latter enzymatic activity was not significantly increased, induced by FA administration.

GR is an enzyme that catalyzes the reduction of glutathione disulfide (GSSG) with electrons provided by NADPH to GSH [71]. This coenzyme is generated in the reaction catalyzed by G6PDH, which is the first step of the oxidative part of the pentose phosphate pathway. NADPH is also used by CYP1A1 and 3A4 for metabolic activation of AFB1 [72] and OTA [73]. Probably due to this need, G6PDH activity increased in the presence of AFB1, OTA, and AFB1 + OTA after both exposure intervals.

ROS interact with protein cysteines, generating sulfenic acid formation, which rapidly interact with nearby cysteine residues, forming inter- and/or intramolecular disulfide bonds. Alternatively, sulfenic acids react with GSH, producing S-glutathionylation. The rate of these biochemical reactions varies in an oxidant-type and cysteine reactivity-dependent manner [74]. Analyzing the variation profiles of GSH and protein thiol levels, we could assume that in the case of our experiment, S-glutathionylation did not occur, and the decrease in protein thiol groups could be due to the disulfide bond formation. After 48 h of exposure, in the AFB1 + OTA + FA sample, the concentration of protein thiol groups increased. FA, being a polyphenolic compound, can donate a hydrogen atom to ROS. Probably, due to this, the oxidation of protein cysteine residues was stopped, and the level of thiol groups increased [75].

## 4. Conclusions

The addition of ferulic acid (FA) in the simultaneous presence of AFB1 + OTA appears to reduce oxidative stress by modulating lipid and protein oxidation. Decreased 4-HNE levels and a significant increase in SOD and GPX-specific activity suggest an adaptive antioxidant response. There are differential kinetics between lipid versus protein oxidation, observed by an initial increase in 4-HNE levels at 24 h of exposure, followed by a decrease in this level at 48 h, together with an increased AOPP levels, which indicates that lipid peroxides are removed more rapidly than oxidized proteins, supporting the idea that protein oxidation is a persistent marker of oxidative stress.

In addition, the variation in the level of GSH and thiol groups indicates that S-glutathionylation does not occur. Although initially the decrease in thiol groups may reflect a formation of disulfide bonds, after 48 h, the addition of FA (AFB1 + OTA + FA) seems to reduce cysteine oxidation, probably by donating hydrogen atoms to ROS and thereby preserving the availability of thiol groups in proteins.

The decrease in relative levels of polyubiquitinated Nrf2 at 48 h along with increased SOD suggests that the addition of FA may induce Nrf2 stabilization, supporting the regulatory antioxidant role of FA and delaying Nrf2 degradation under chronic oxidative stress. The biochemical changes observed with single and/or concomitant administration of AFB1, OTA, and FA suggest that FA may partially counteract mycotoxin-induced oxidative damage through some effects, such as the modulation of protein oxidation markers, which require further investigation for long-term implications.

## 5. Materials and Methods

### 5.1. Cell Line

In vitro experiments were performed on C2BBe1 cells (ATCC CRL-2102), a subclone of the Caco-2 cell line, which is derived from human colonic epithelial tissue from a 72-year-old Caucasian male adult with colorectal adenocarcinoma and isolated from the apical zone of the intestinal villi. The adherent cells were grown in monolayer in DMEM media (31600-083, Life Technologies Gibco, Paisley, UK), supplemented with 1.5 g/L sodium bicarbonate (S5761, Sigma-Aldrich, St. Louis, MO, USA), 0.01 mg /mL transferrin (T8158, Sigma-Aldrich, St. Louis, MO, USA), 1% antibiotic mix (penicillin, streptomycin and amphotericin) (A5955; Sigma-Aldrich, St. Louis, MO, USA), and 10% fetal bovine serum (10270-106, origin South America, Gibco, by Life Technologies, Carlsbad, CA, USA) and were incubated at 37 °C in a humid atmosphere with 5% CO_2_.

### 5.2. Cells’ Treatment and MTT Assay

To evaluate the cell viability, 20,000 cells/mL were seeded in each well of a 96-well plate and left to adhere overnight. Several concentrations of the tested compound were used (Table A1). After 24, 48, and 72 h after the treatment, 80 μL of 1 mg /mL MTT solution (M2128, Sigma-Aldrich, St. Louis, MO, USA) was added to each well, and the plate was incubated for 2 h at 37 °C. The formazan crystals were solubilized in 150 μL isopropanol (33539, Sigma-Aldrich, St. Louis, MO, USA) and mixed thoroughly. The absorbance was read at 595 nm using a FlexStation3 spectrophotometer (Molecular Devices LLC, San Jose, CA, USA). Control was considered with 100% viability. After analyzing the data (Figure A1 and Figure A2), we decided to use the following doses for the next analyses: for AFB1 25 μM, for OTA 10 μM, and for FA 10 μM. Thus, 5 experimental groups resulted: AFB1 25 μM (AFB1), OTA 10 μM (OTA), AFB1 25 μM + OTA 10 μM (AFB1 + OTA), AFB1 25 μM + OTA 10 μM + FA 10 μM (AFB1 + OTA + FA), and FA 10 μM (FA).

For the next biochemical analyses, the cells were seeded at a density of 10^5^ cells/ mL in 75 cm^2^ flasks and allowed to adhere for 24 h. Then, they were exposed to the chosen doses either alone or in combinations, for 24 h and 48 h, respectively. Untreated cells were used as the Control group.

Before the biochemical analysis, the cell lysate was prepared, thus after trypsinization (T4709, Sigma-Aldrich, St. Louis, MO, USA), the C2BBe1 cells were centrifuged for 5 min at 1500× *g* at RT, and the pellets were washed and resuspended in 350 μL phosphate buffer saline (PBS, 18912-014, Gibco, Grand Island, NY, USA). The cells’ lysates were obtained by ultrasonication using a UP50H sonicator (Hielscher Ultrasound Technology, Teltow, Germany) set at one cycle and 80% amplitude, 3 times on ice for 30 s each with 1 min between sonication. The total protein extract was centrifuged at 3000× *g* for 10 min at 4 °C, and the clear supernatants were collected. The protein concentrations for each condition were quantified by the Bradford method (B6916, Merck, St. Louis, MO, USA), using a calibration curve with bovine serum albumin (BSA) as standard, ranging from 0 to 1.5 µg/mL protein [76].

### 5.3. Assessment of Oxidative Stress and Lipid Peroxidation

#### 5.3.1. Detection of Malondialdehyde (MDA) Level

Malondialdehyde is a marker of lipid peroxidation and was assessed according to the method described by Del Rio (2003) [77], using 2-thiobarbituric acid (TBA, T5500, Sigma-Aldrich, St. Louis, MO, USA). Briefly, 20 μL of clear cell lysate was mixed with 70 µL of 0.1 N HCl (30721, Fluka, Charlotte, NC, USA) and incubated for 20 min at room temperature. Subsequently, a volume of 90 μL of 0.025 M thiobarbituric acid (TBA) was added, and the samples were incubated for 65 min at 37 °C. In the end, a volume of 40 μL of PBS was added. MDA-TBA adduct fluorescence was measured at 520 nm excitation and 549 nm emission, using a FlexStation 3 multi-reader. MDA concentrations were quantified via a calibration curve ranging between 0.05 and 0.5 μM, using 1,1,3,3-tetraethoxypropane as the standard. Lipid peroxidation of the samples was assessed by normalizing MDA levels to protein concentration.

#### 5.3.2. Advanced Oxidation Protein Products (AOPPs)

Advanced oxidation protein products (AOPPs) were quantified by the method described by Witko-Sarsat et al. (1992) [78], a spectrophotometric method based on the chloramine’s ability to oxidize potassium iodide. Thus, in a 96-well plate, 10 µL potassium iodide (1.16 M solution prepared in distilled water) was added over 200 µL of the sample and incubated with gentle shaking for 5 min at room temperature. At the end of the incubation time, 20 µL glacial acetic acid (A/0400/PB15, Fisher Chemical, Pittsburgh, PA, USA) was added, and the plate was shaken for 30 s at room temperature, after which the absorbance was read at 340 nm on a FlexStation3 multireader spectrometer. The results obtained were expressed in AOPP micromoles/mg protein, by interpolating the optical densities obtained from the samples on a calibration curve with chloramine T (857319, Sigma-Aldrich, St. Louis, MO, USA) [5–100 µM], where the stock solution of 100 µM chloramine T was prepared in PBS containing BSA 1 mg/mL. Subsequently, the AOPP micromole values were reported to the corresponding protein concentration for each sample.

#### 5.3.3. Western Blot

For the relative quantification of Nrf2 and 4-HNE in clear cell lysate samples, Western blotting was used following the method previously described by Popescu et al. (2021) [79]. Thus, Western Breeze Chromogenic Western Blot Immunodetection Kit Version F June 4, 2003 June 4, 2003 IM-2004 (Invitrogen), anti Nrf2 (rabbit) primary antibody (H-300) (SC 13032; Santa Cruz Biotechnology, Heidelberg, Germany) at 1:250 dilution, anti 4-HNE (rabbit) polyclonal primary antibody at 1:250 dilution (bs 6313R; Bioss Antibodies, Woburn, MA, USA) were used, following the manufacturers’ instructions.

Protein bands were visualized using the ChemiDoc MP imaging system (Bio-Rad, Hercules, CA, USA) and quantified with ImageLab software 5.1 (Bio-Rad). To ensure equal protein loading, each sample was normalized to β-actin expression (mouse monoclonal antibody A1978, Sigma-Aldrich, 1:1000).

### 5.4. Non-Enzymatic Antioxidant Defense

#### 5.4.1. Quantification of Reduced Glutathione (GSH) Concentration

The concentration of intracellular GSH was determined using a colorimetric Glutathione Assay Kit (CS0260, Sigma-Aldrich, St. Louis, MO, USA), according to the manufacturer’s instructions. Briefly, clear cell lysates were deproteinized with 5% sulfosalicylic acid (1:1 *v*/*v*) and centrifuged at 10,000× *g* for 10 min at 4 °C to remove precipitated proteins. Subsequently, a volume of 10 μL of clear supernatant was mixed with 150 μL working mixture made from 8 mL of buffer solution (100 mM potassium phosphate buffer, pH 7; 1 mM EDTA—E5391, Sigma-Aldrich, St. Louis, MO, USA) and 228 μL of 1.5 mg/mL 5,5′-Dithiobis(2-nitrobenzoic acid) (DTNB—D8130, Sigma-Aldrich, St. Louis, MO, USA) and incubated for 10 min at room temperature. The quantity of 5-thio-2-nitrobenzoic acid (TNB) yellow products was measured spectrophotometrically at 405 nm, using a calibration curve of GSH in the range 3.125–50 μM. The final concentration of GSH was reported to protein concentration for each sample and expressed as nanomoles GSH/mg protein.

#### 5.4.2. Thiol Groups

Thiol group quantification was performed following the method described by Ellman (1959) [80] and adapted from Riener et al. (2002) [81]. The principle of this method is based on the reaction between thiol groups (-SH) and 4,4′-dithiodipyridine (DTDP), forming 4-pyridyldithio derivatives (R-S-S-Pyr) that absorb at 324 nm. These derivatives can further react with other thiol groups, generating symmetrical disulfide-bridged compounds (R-S-S-R).

Thus, 100 µL of 20% trichloroacetic acid (TCA) was added to 100 µL of the appropriately diluted sample, homogenized, and the mixture was kept on ice for 10 min, after which it was centrifuged at ~9000× *g* for 10 min at 4 °C. After centrifugation, the supernatant was removed, and the pellet was solubilized in 20 µL 1 M NaOH (30620, Fluka, Charlotte, NC, USA), after which 730 µL 0.4 M Tris buffer (T6066, Sigma-Aldrich, St. Louis, MO, USA), pH 9, was added. After the mixture was homogenized, 30 µL of 4 mM 4,4′-dithiodipyridine (DTDP) was added and incubated for 5 min in the dark at room temperature, after which the absorbance was read at 324 nm using a FlexStation3 multileader spectrophotometer. In parallel, a blank reaction and a calibration curve of N-acetyl cysteine [10–100 µM] from a 3 mM stock solution were performed. The absorbance values obtained were interpolated to the N-acetyl cysteine curve, and the results were expressed in micromoles relative to the corresponding amount of protein (micromoles –SH/mg protein).

### 5.5. Enzymatic Antioxidant Defense

#### 5.5.1. Catalase

Catalase (EC 1.11.1.6) activity was determined by monitoring the decrease of H_2_O_2_ absorbance at 240 nm, according to the method described by Beers and Sizer [82]. The reaction kinetics was registered spectrophotometrically for 1 min after mixing the cell lysate with 0.059 M H_2_O_2_ and 0.1 M K_2_HPO_4_/KH_2_PO_4_ buffer, pH 7.1 (60355, Fluka, Charlotte, NC, USA). Catalase specific activity was expressed in U/mg protein, where one U represents the amount of enzyme that catalyzed the conversion of one micromole H_2_O_2_ in 1 min at room temperature, and divided by 43.6, where 43.6 represents the molar extinction coefficient of H_2_O_2_ at 240 nm (ε_H2O2_ = 43.6 × 10^3^ M^−1^ cm^−1^).

#### 5.5.2. Superoxide Dismutase

Superoxide dismutase (SOD) (EC 1.15.1.1) activity was measured according to the method described by Paoletti et al. (1986) [83], which is based on the oxidation of NADPH by superoxide anions. Thus, superoxide anions were generated from molecular oxygen by adding a triethanolamine: diethanolamine buffer (for each 100 mM, pH 7.4) containing MnCl_2_-EDTA (100 mM/50 mM) and 2-mercaptoethanol (100 mM) to each sample. Following the addition of 7.5 mM NADPH, the decrease in absorbance at 340 nm was monitored for 10 min at 37 °C. A blank without the sample was analyzed in parallel. SOD specific activity was expressed as U/mg protein, where one unit (U) represents the enzyme amount required to inhibit the NADH oxidation rate by 50% compared to the control sample, and the value was multiplied by 1.7122 and the sample dilution factor, where 1.7122 represents the molar extinction coefficient (ε) of NADH at 340 nm.

#### 5.5.3. Glutathione Peroxidase

Glutathione peroxidase (EC 1.11.1.9) activity was assessed using a method described by Beutler (1984) [84], using 7 mM tert-butyl-hydroperoxide and 2 mM NADPH as substrates. The oxidation of NADPH to NADP^+^ was monitored by measuring the absorbance decrease at 340 nm for 5 min. One unit (U) of glutathione peroxidase activity was defined as the amount of enzyme required to catalyze the oxidation of 1 μmol of NADPH per minute at 25 °C, multiplied by the molar extinction coefficient (ε) of NADPH of 6.22 × 10^3^ M^−1^ cm^−1^. Specific activity was expressed as U/mg protein.

#### 5.5.4. Glutathione S-Transferase

Glutathione S-transferase (GST) (EC 2.5.1.18) activity was measured using the method described by Habig et al. (1974) [85]. The enzymatic reaction involves the conjugation of GSH with an electrophilic substrate represented by 1-chloro-2,4-dinitrobenzene (CDNB) and monitoring the increase in absorbance at 340 nm for 1 min. A blank was made in parallel for each sample. Thus, 200 µL of 0.1 M H_2_HPO_4_/KH_2_PO_4_ buffer pH 7, 20 µL 25 mM CDNB, and 100 µL 20 mM GSH were added to 630 µL of the corresponding diluted sample or distilled water (for the blank). After homogenization of the reaction mixture, the optical density was read at 340 nm, at 25 °C, continuously for 5 min. The results were expressed as GST specific activity (U/mg), where one unit represents the amount of enzyme required to form 1 micromole of GS-CDNB per minute at 25 °C, multiplied by the molar extinction coefficient (ε) of CDNB of 9.6 × 10^3^ M^−1^ cm^−1^.

#### 5.5.5. Glutathione Reductase

Glutathione reductase (EC 1.6.4.2) activity was measured according to the method described by Goldberg and Spooner (1986) [86] by monitoring the decrease in absorbance at 340 nm due to NADPH oxidation. Thus, 50 µL of 0.1 M H_2_HPO_4_/KH_2_PO_4_ buffer pH 7, 50 µL 2 mM NADPH, and 20 µL 33 mM GSSG were added to 855 µL of the corresponding diluted sample. In parallel, a blank was made with distilled water in which the volume of GSSG was not added. The results were expressed as glutathione reductase specific activity (U/mg), where one unit represents the amount of enzyme needed to catalyze the oxidation of one µmole NADPH in 1 min at 25 °C, multiplied by the molar extinction coefficient (ε) of NADH of 6.22 × 10^3^ M^−1^ cm^−1^.

#### 5.5.6. Glucose-6-Phosphate Dehydrogenase

Glucose 6-phosphate dehydrogenase (EC 1.1.1.49) activity was assessed by measuring the increase in NADPH absorbance at 340 nm [87]. The reaction was performed using 670 µL 50 mM HEPES buffer solution, pH 7.5, 20 µL 40 mM glucose-6-phosphate, and 50 µL sample or distilled water for the blank. After reaction homogenization, 10 µL of 30 mM NADP^+^ prepared in 1% Na_2_CO_3_ was added only to the sample reaction (S7795, Sigma-Aldrich, St. Louis, MO, USA), after which the optical density was continuously read at 340 nm for 5 min at 25 °C. The results were expressed as glucose 6-phosphate dehydrogenase specific activity (U/mg), where one unit represents the amount of enzyme required to generate 1 µmol NADPH in 1 min at 25 °C, multiplied by the molar extinction coefficient (ε) of NADH of 6.22 × 10^3^ M^−1^ cm^−1^.

### 5.6. Statistical Analysis

Since all results depended on two independent factors, the type of compound and the exposure time (24, 48, and 72 h), statistical analysis was performed using two-way ANOVA. Post hoc comparisons among all groups were carried out using Tukey’s test. Statistical significance (*p*-value) is presented only for comparisons where *p* < 0.05. For each analysis, each of the biological replicates was performed in triplicate. Also, the statistical power for the tests used, calculated post hoc, was greater than 0.89. All analyses and graphs were generated using R (version 4.4.1).

## Data Availability

The original contributions presented in this study are included in the article. Further inquiries can be directed to the corresponding author.

## Appendix A

**Table A1:** Experimental conditions used in the present study.

Condition Name	Concentration [μM]		
	OTA	AFB1	FA
Control AFB1	-	-	-
AFB1 0.5	-	0.5	-
AFB1 5	-	5	-
AFB1 25	-	25	-
Control OTA	-	-	-
OTA 1	1	-	-
OTA 5	5	-	-
OTA 10	10	-	-
Control FA	-	-	-
FA 10	-	-	10
FA 40	-	-	40
Control AFB1 + OTA	-	-	-
OTA 1 + AFB1 0.5	1	0.5	-
OTA 5 + AFB1 5	5	5	-
OTA 10 + AFB1 25	10	25	-
OTA 1 + AFB1 25	1	25	-
OTA 10 + AFB1 0.5	10	0.5	-
Control OTA + AFB1 + FA	-	-	-
OTA 1 + AFB1 0.5 + FA 10	1	0.5	10
OTA 1 + AFB1 0.5 + FA 40	1	0.5	40
OTA 5 + AFB1 5 + FA 10	5	5	10
OTA 5 + AFB1 5 + FA 40	5	5	40
OTA 10 + AFB1 25 + FA 10	10	25	10
OTA 10 + AFB1 25 + FA 50	10	25	40
OTA 1 + AFB1 25 + FA 10	1	25	10
OTA 1 + AFB1 25 + FA 40	1	25	40
OTA 10 + AFB1 0.5 + FA 10	10	0.5	10
OTA 10 + AFB1 0.5 + FA 40	10	0.5	40

OTA—ochratoxin A; AFB1—aflatoxin A1; FA—ferulic acid.
